# Estrogen represses gene expression through reconfiguring chromatin structures

**DOI:** 10.1093/nar/gkt586

**Published:** 2013-07-01

**Authors:** Hatice Ulku Osmanbeyoglu, Kevin N. Lu, Steffi Oesterreich, Roger S. Day, Panayiotis V. Benos, Claudia Coronnello, Xinghua Lu

**Affiliations:** ^1^Department of Biomedical Informatics, University of Pittsburgh, Pittsburgh, PA, USA, ^2^Department of Computer Science, University of Pittsburgh, Pittsburgh, PA, USA, ^3^Department of Pharmacology and Chemical Biology, University of Pittsburgh, Pittsburgh, PA, USA, ^4^Women’s Cancer Research Center, University of Pittsburgh, Pittsburgh, PA, USA, ^5^Department of Computational and Systems Biology, University of Pittsburgh, Pittsburgh, PA, USA and ^6^Fondazione Ri.MED, Palermo, Italy

## Abstract

Estrogen regulates over a thousand genes, with an equal number of them being induced or repressed. The distinct mechanisms underlying these dual transcriptional effects remain largely unknown. We derived comprehensive views of the transcription machineries assembled at estrogen-responsive genes through integrating multiple types of genomic data. In the absence of estrogen, the majority of genes formed higher-order chromatin structures, including DNA loops tethered to protein complexes involving RNA polymerase II (Pol II), estrogen receptor alpha (ERα) and ERα-pioneer factors. Genes to be ‘repressed’ by estrogen showed active transcription at promoters and throughout the gene bodies; genes to be ‘induced’ exhibited active transcription initiation at promoters, but with transcription paused in gene bodies. In the presence of estrogen, the majority of estrogen-induced genes retained the original higher-order chromatin structures, whereas most estrogen-repressed genes underwent a chromatin reconfiguration. For estrogen-induced genes, estrogen enhances transcription elongation, potentially through recruitment of co-activators or release of co-repressors with unique roles in elongation. For estrogen-repressed genes, estrogen treatment leads to chromatin structure reconfiguration, thereby disrupting the originally transcription-efficient chromatin structures. Our *in silico* studies have shown that estrogen regulates gene expression, at least in part, through modifying previously assembled higher-order complexes, rather than by facilitating *de novo* assembly of machineries.

## INTRODUCTION

Estrogen is essential for the development and function of the female reproductive system, and is a known potent mitogen in breast cancer (1,2). The effects of estrogen are mediated through the alpha and beta estrogen receptors (ERα and ERβ), which are canonical examples of a large family of transcription regulators referred to as nuclear receptors. It is widely believed that, when bound by their corresponding ligands, nuclear receptors bind to DNA in a sequence-specific manner and facilitate assembly of transcription machineries at the sites. However, this view cannot explain the phenomenon that almost an equal number of genes can be repressed or induced by estrogen-bound ERα (3). While there is an extensive body of research studying ERα as a transcription activator—see review articles (4,5)—few studies concentrate on the mechanisms of ERα-mediated transcriptional repression (6–10), and the majority of those that do concentrate on a small number of estrogen-responsive genes. Therefore, the mechanisms by which estrogen represses gene expression at a genome scale remain largely unclear. Because many nuclear receptors play dual regulatory roles, a better understanding of the mechanism of ERα-mediated gene repression would shed light on general mechanisms by which a transcription regulator exerts dual inductive and repressive effects.

The emergence and application of high-throughput technologies enable studies inspecting different aspects of transcription processes on a genome-wide scale. For instance, microarray technology measures overall mRNA, whereas global run-on sequencing (GRO-seq) measures *de novo* transcriptional activities. Chromatin immunoprecipitation (ChIP) followed by high-throughput DNA sequencing (ChIP-seq) enables genome-wide profiling of the protein–DNA interaction of transcription factors, co-regulators, RNA polymerase II (Pol II) and histone-modification markers, while chromatin interaction analysis with paired-end tag sequencing (ChIA-PET) and other techniques (11–14) captures long-range chromatin interactions on a genome-wide scale.

In this study, we sought to investigate the mechanisms of estrogen-mediated transcription regulation by integrating publically available genome-scale data sets collected in the absence and presence of estrogen (15–21). Through dissecting the diverse data sets from different angles, we derived an extensive picture of ERα-mediated transcription machinery, particularly with respect to the involvement of higher-order chromatin structures and their distinct responses to estrogen between estrogen-induced and estrogen-repressed genes. Our analyses resulted in new findings with respect to both baseline transcription and ligand-mediated transcription of estrogen-regulated genes. These findings further lead to a novel hypothesis for a general mechanism for gene repression.

## MATERIALS AND METHODS

### Identification of consensus estrogen-responsive genes

The consensus estrogen-responsive genes were identified based on a ranked-product meta-analysis across four independently published data sets (GSE3834, GSE9936, GSE11324 and GSE5840—Affymetrix GeneChip Human Genome U133 Plus 2.0 platform), which investigated the effect of estrogen treatment on gene expression in MCF-7 cells at early (3–4 h) time points (22). We further filtered out genes with small mean and standard deviation, which would lead to a low signal-to-noise ratio. We selected genes that contain a single RefSeq transcription-starting site (TSS) annotation to simplify analysis.

### ChIP-seq, GRO-seq and ChIA-PET data sets and preprocessing

ChIA-PET data for MCF-7 cells (preprocessed) were obtained from the originally published Supplementary Data (15,16,23). We merged results of IHM001F and IHH015F large-scale ChIA-PET analysis (15) using supplementary files of the original work (15). Processed Pol II ChIA-PET data were obtained from authors of the original work (23). Preprocessed ChIP-seq Pol II, transcription factor (TF), co-regulator and histone-marker data for MCF-7 were downloaded from the Nuclear Receptor Cistrome Database, (21) where the peaks were called by the model-based analysis of ChIP-Seq (MACS) method (26), with *P*-value cutoff 10^−^^5^. Mapped GRO-seq reads (at 0 and 40 min) were downloaded from the GEO (GSE27463).

### Consensus ERα cistrome

We collected a total of four ChIP-seq data sets for ERα (17–20) that profiled MCF-7 in the absence and presence of ligand. Because there was a large variation of ERα-binding sites across different MCF-7 studies, we merged overlapping binding sites in at least two studies to form a consensus ERα cistrome using the completeMOTIFs pipeline (24). This approach allowed us to combine the results of several studies to provide a global picture of ERα-binding sites.

### Consensus FoxA1 cistrome

From the MCF-7 cell line, we collected three ChIP-seq data sets for FoxA1 (17,25,26) in the absence of ligand, as well as two ChIP-seq data sets for FoxA1 (17,25) treated with estrogen. We merged overlapping binding sites in at least two studies to form a consensus FoxA1 cistrome in the absence of ligand using the completeMOTIFs pipeline (24). We took overlapping binding sites (at least 1 bp) from two studies of ligand to form a FoxA1 cistrome in the presence of ligand.

### Genome annotations

Genome annotations were downloaded from the human genome Build 36 (hg18 assembly) of the UCSC Genome Browser (www.ucsc.org). Gene definitions were given by the RefSeq genes track. We have considered only the RefSeq genes that have one annotated TSS. When visualizing experiments with the UCSC Genome Browser, we used human genome Build 37 (hg19 assembly).

### Pol II ChIP-seq meta-gene profiles

The average Pol II ChIP-seq profile across genes, a ‘metagene’ profile (27), was plotted by aligning genes at the first and last nucleotides of the annotated transcripts and scaling the sequencing tags as follows. The total sequence tag counts were directly used for the promoter (0.5 kb upstream of the TSS to 0.5 kb downstream) and the 3′-end (0.5 kb upstream of the transcription end site (TES) to 0.5 kb downstream) of transcripts. To account for variable gene sizes, a signal between 0.5 kb downstream of the TSS to 0.5 kb upstream of the gene end was represented by 1000 values obtained by cubic spline interpolation. The resulting tags of a gene were then scaled to 100 equally sized bins (with average tags in each bin), so that all genes appeared to have the same length. All profiles were plotted on a normalized read per million (RPM) basis.

### GRO-seq meta-gene profiles

To show the average GRO-seq profiles across genes, we plotted a ‘metagene’ profile (27). Genes were aligned at the first and last nucleotides of the annotated transcripts, and sequencing tags were scaled in the same fashion as described in the previous subsection. The total sequencing tag counts were directly used for the promoter (1 kb upstream of the TSS to 1 kb downstream) and the 3′-end (1 kb upstream of the TES to 1 kb downstream) of transcripts. All profiles were plotted on a normalized RPM basis. The transcription-starting rate on the sense (or antisense) strand of a gene was calculated as the total RPM near the TSS region (−300 to +300 bp).

### microRNA analysis

The target prediction analysis was performed by using ComiR (28), a newly developed algorithm that is designed to predict the targets of a set of microRNAs (miRNAs). ComiR incorporates the miRNA expression level in the thermodynamic binding model and thus improves the prediction of existing algorithms. It then combines the improved predictions of four target prediction tools using a support vector machine trained on Drosophila Ago1 immunoprecipitation data. We used ComiR to compute the gene probabilities associated with each single miRNA, and we considered as targets those genes with a ComiR probability score >0.8.

### Statistical analysis

The statistical significance of the difference between gene groups was assessed using the *t*-test and the chi-squared test using R. To gauge the significance of spatial correlations between promoters of estrogen-responsive genes with histone markers, we used the GenometriCorr package (29), which assesses permutation to (*n* = 500) to create a distribution of genomic distances that would be expected if the markers were uncorrelated.

## RESULTS

### Identification of early estrogen-responsive genes by meta-analysis

We used the results from a recent meta-analysis of estrogen response in MCF-7 breast cancer cells (22) that identified a set of early estrogen-responsive genes. From this set, we selected genes that have a single TSS according to annotations from RefSeq (30), leading to a total of 748 estrogen-responsive genes, including 429 estrogen-induced and 319 estrogen-repressed genes (Supplementary Table S5). For the estrogen-induced genes, the signals at the probe level for 210 genes were categorized as ‘present’ in the absence of estrogen, and those for the remaining 219 genes were categorized as ‘absent’. In the absence of estrogen, the expression value of estrogen-repressed genes was higher than that of estrogen-induced genes. This subset of estrogen-responsive genes in the human genome should be sufficiently representative of estrogen-responsive genes to allow for insight into estrogen-mediated transcription regulation.

### The status of transcription machineries in the absence of estrogen

To infer the status of transcription machineries assembled at the estrogen-responsive genes in the absence of estrogen, we performed integrative analyses of a large number of the genome-scale data sets collected from MCF-7 cells, including ChIP-seq data sets for Pol II, ERα, ERα-pioneer factors and ChIA-PET data sets for Pol II and ERα (see Supplementary Table S1 for the complete list of data sets).

#### Pol II occupancy at promoters

**Figure 1.**
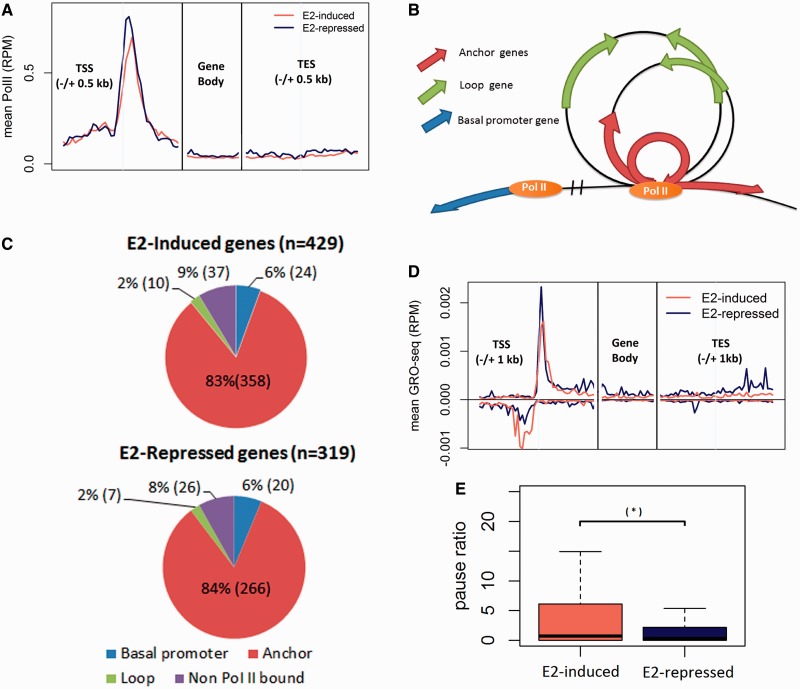
Transcription states and chromatin complexes in the absence of estrogen. (**A**) Composite (meta-gene) profiles of Pol II ChIP-seq of estrogen-responsive genes, presented as RPM. Profiles for promoter and 3′ end were aligned at TSS and TES respectively; profiles for gene bodies were scaled. (**B**) Annotation of genes based on their relative position to the Pol chromatin complex. (**C**) Distribution of estrogen-responsive genes in terms of their relative position to Pol II complexes. (**D**) Meta-gene profiles of GRO-seq of Pol II bound anchor genes, presented as RPM. Profiles for promoter and 3′ end were aligned at TSS and TES, respectively; profiles for gene bodies were scaled. GRO-seq reads aligned to RefSeq TSSs in both sense and antisense directions relative to the direction of the gene. (**E**) Boxplots show the comparison of pause ratio (TSS/gene body) for estrogen-repressed genes (blue) and estrogen-induced genes (coral) as determined by GRO-seq in the absence ligand.

Using the Pol II ChIP-seq data set by Li *et al.* (23), we performed meta-gene analysis to compare Pol II occupancy at estrogen-induced and estrogen-repressed genes (Figure 1A). Both gene sets showed strong peaks of Pol II-binding at the promoter regions of the meta-genes, and revealed no significant difference (*t*-test *P* = 0.7) in terms of the normalized total sequence tag counts near TSS (±500 bp) between the two sets of genes. Because Pol II was ubiquitously present at the promoters of estrogen-responsive genes, and because estrogen was not required for Pol II recruitment to these promoters, we conclude that Pol II occupancy in the absence of estrogen is not the key factor determining the distinct transcriptional behaviors of the estrogen-induced and estrogen-repressed genes.

#### Pol II-associated chromatin interaction complexes.

Observing the presence of Pol II at the estrogen-responsive genes motivated us to investigate whether DNA-bound Pol II participate in higher-order chromatin structures because it is known that physical contacts between regulatory factors through local DNA looping cause higher-order structures that affect gene-expression regulation (15,23,31). We studied the Pol II ChIA-PET data by Li *et al.* (23) to investigate higher-order chromatin structures involving Pol II (‘Pol II complexes’) at estrogen-responsive genes.

The simplest form of a higher-order chromatin structure is a single loop of DNA tethered to a protein complex, and it is common to observe multiple loops and anchor regions (the DNA region in contact with the protein complex) in a chromatin complex. The diagram at Figure 1B illustrates the way a gene can be categorized with respect to a chromatin complex. A gene is considered an ‘anchor gene’ if its TSS is within ±5 kb of a DNA–protein anchor region (23); otherwise, it is considered a ‘loop gene’. Such a categorization is of interest because being an ‘anchor gene’ brings a gene close to Pol II, the center of transcription action.

Among 748 estrogen-responsive genes, 641 (86%) were located within 563 Pol II complexes, indicating that some Pol II complexes enclosed more than one estrogen-responsive gene. Further inspection showed that the majority—624 out of the 641 genes (97%)—were located in the anchor regions of the 552 Pol II complexes; of these, 358 were estrogen-induced genes and 266 were estrogen-repressed genes (Figure 1C). The rest of this report will focus on these estrogen-responsive anchor genes to study the impact of formation or disruption of higher-order chromatin structures on regulating their expression.

#### ERα and pioneer factors within the chromatin complexes.

The presence of Pol II complexes at the estrogen-responsive genes raised an interesting question: are ERα and its pioneer factors involved in the formation of these complexes in the absence of estrogen? From the results of four ERα ChIP-seq studies (17–20) in MCF-7 cells before estrogen treatment, we identified a set of 18 212 consensus ERα-binding sites. We then investigated which of the observed ERα-binding sites were located within or close to the Pol II complex regions containing estrogen-responsive genes.

The majority (434/552 = 79%) of the Pol II complexes containing estrogen-responsive anchor genes contained ERα-binding sites, averaging 9.1 sites per complex. Most of them had at least one site within an anchor region (413/434 = 95%). Table 1 shows the distribution of complexes containing estrogen-induced and estrogen-repressed genes, as well as the percentage of complexes containing ERα-binding sites. A Jaccard test (29) showed that, in the absence of estrogen, the ERα sites were significantly enriched in the Pol II complexes (*P* < 0.01).

**Table 1.** The distribution of Pol II complexes where estrogen-induced and estrogen-repressed genes reside and their relationship to ERα and pioneer factor binding sites inside the anchor region of complexes

Number	Complexes containing estrogen-induced genes (%)	Complexes containing estrogen-repressed genes (%)
All Pol II complexes	344	238
ERα	248 (72.1%)	194 (81.5%)
FoxA1	199 (57.8%)	187 (78.6%)
AP2γ	286 (83.1%)	222 (93.3%)
PBX1	271 (78.8%)	211 (88.7%)
GATA3	235 (68.3%)	193 (81.1%)

The table shows the number of complexes—where estrogen-induced and estrogen-repressed genes reside—that have ERα and pioneer factor binding inside their anchor regions.

We further investigated whether the well-known ERα-pioneer factor, forkhead box A1 (FoxA1), as well as other putative ERα-pioneer factors, including pre-B-cell luekemia trnascription factor 1 (PBX1), activating protein 2 gamma (AP2γ) and trans-acting T-cell-specific transcription factor (GATA3), were also enriched in the Pol II complexes to facilitate ERα binding in these regions. We identified the binding sites for these factors from respective ChIP-seq data (17,25,26,32,33) collected from MCF-7 cells in the absence of estrogen (Table 1). Jaccard tests (29) showed that the binding sites of each the above factors were significantly enriched (*P* < 0.01) in the anchor regions. Most ERα-binding sites (3045 out of 3950; 77%) co-occurred with at least one pioneer factor inside the Pol II complexes (Supplementary Figures S1 and S2).

#### Histone-modification markers

Epigenetic modifications, particularly histone-modification markers, reflect another aspect of the status of transcription machineries at promoters. Some of these markers indicate ‘active promoters’, while others indicate ‘inactive promoters’ (34). Using the GenometriCorr package (29), we analyzed the histone-modification-marker data from MCF-7 cells (17,35) to assess whether they exhibited significant spatial correlation with the promoters (±1 kb around TSS) of the estrogen-responsive genes. The results (see Supplementary Table S2) show that, in the absence of estrogen, the promoters of both estrogen-induced and -repressed genes were significantly enriched with the histone-modification markers commonly associated with active promoters, including H3K4me1, H3K4me3, H3K9ac and H3K14ac. There was no significant difference in histone-marker distributions between estrogen-induced and estrogen-repressed genes.

#### Transcription activity

Next, we investigated the transcription activity of estrogen-responsive genes in the absence of estrogen, using the results of a GRO-seq experiment performed in MCF-7 (16). GRO-seq detects *de novo* transcription activities of genes on a genome-wide scale, thus providing a ‘map’ of the position and direction of transcription activities. We performed meta-gene analysis of the transcription activities in the region between 1 kb upstream of the TSS and 1 kb downstream of the TES. Figure 1D reveals two GRO-seq peaks within the promoters, along both sense and antisense strands of both estrogen-induced and estrogen-repressed genes, indicating that transcription was actively initiated at the TSSs of these genes. On the sense strand, there was no significant difference (*t*-test, *P* = 0.57) in transcription initiation rate (the total reads within the vicinity of ±300 bp of TSS) between the estrogen-induced and estrogen-repressed genes. On the antisense strand, we observed significantly more reads for the estrogen-induced genes in comparison with the estrogen-repressed genes (*t*-test, *P* < 10^−^^3^). The function of antisense transcripts is unknown, but their existence suggests that DNA assumes an open chromatin structure permissive to transcription activity by Pol II (36–38).

We next compared the transcription activities within the gene bodies (300 bp downstream TSS to TES) of the estrogen-responsive genes. Estrogen-repressed genes had more reads in the body region than did estrogen-induced genes (*t*-test *P* < 10^−^^3^). We calculated the pause ratio of each gene, defined as the normalized total reads in the vicinity of a TSS (±300 bp) over that in the corresponding gene body, and compared the results from estrogen-induced and estrogen-repressed genes. Figure 1E shows that the pause ratio of the estrogen-induced genes was significantly higher (*t*-test *P* < 10^−^^2^). The results suggest that, although there was active transcription initiation at the promoter of all estrogen-responsive genes in the absence of estrogen, the transcription of the estrogen-induced genes failed to progress into gene bodies, thus leading to a low level of full-length transcripts; for the estrogen-repressed genes, coordinated transcription across promoters and genes bodies led to full-length transcripts before estrogen treatment.

### Status of transcription machineries in the presence of estrogen

#### Pol II occupancy and transcription activity

**Figure 2.**
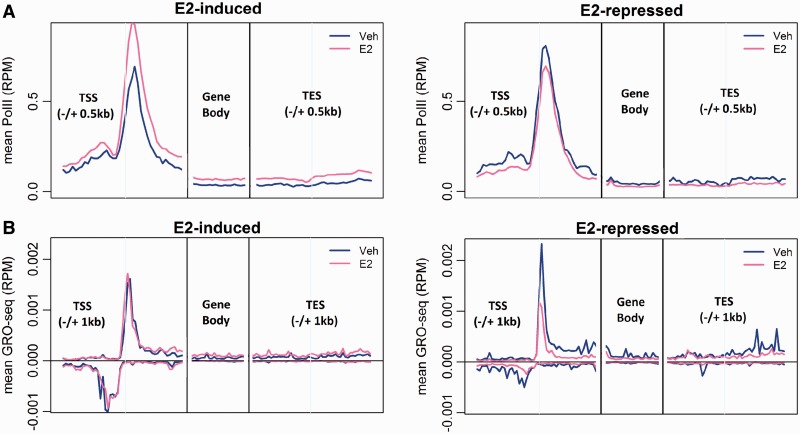
Comparison of transcription states of the estrogen-induced and estrogen-repressed genes. (**A**) Comparison of meta-gene profiles of Pol II ChIP-seq of estrogen-induced and estrogen-repressed genes in the absence (blue) and in the presence of estrogen (pink), presented as RPM. (**B**) Comparison of *de novo* transcription of estrogen-induced and estrogen-repressed genes in the absence (blue) and in the presence of estrogen (pink) determined by GRO-seq for the estrogen-induced (left) and estrogen-repressed (right) genes.

After estrogen treatment, Pol II occupancy (18) was found to be significantly increased (paired *t*-test, *P* < 10^−^^19^) and significantly decreased (*P* < 10^−^^10^) for the estrogen-induced and estrogen-repressed genes, respectively (Figure 2A).

We further studied the impact of estrogen treatment on *de novo* transcription rates (Figure 2B) using the GRO-seq data (16). For the estrogen-induced genes, the transcription rate at promoter regions did not change significantly (paired *t*-test, *P* = 0.16), but the transcription rate in the gene body (sense strand) increased significantly (paired *t*-test, *P* < 10^−^^19^), and the pause ratio was significantly decreased (paired *t*-test, *P* = 0.0015). We found no significant change in the antisense transcription before and after estrogen treatment (paired *t*-test, *P* = 0.85). These results indicate that estrogen mainly acted to enhance the elongation of transcription of the estrogen-induced genes.

For the estrogen-repressed genes, estrogen reduced the transcription level in both promoter (paired *t*-test, *P* = 0.012) and gene body (*P* < 10^−^^6^) regions. The comparison of pause ratios before and after estrogen treatment showed no statistical difference (paired *t*-test, *P* = 0.21). However, antisense transcription significantly decreased in the presence of estrogen (paired *t*-test, *P* < 10^−^^5^). These results suggest that estrogen effected the transcription repression, at least in part, by suppressing transcription initiation.

#### Histone-modification markers

We next analyzed the impact of estrogen treatment on histone modification at the promoters of estrogen-responsive genes (17). As shown in Supplementary Table S2, estrogen treatment did not significantly change the distributions of histone-modification markers at either estrogen-induced or estrogen-repressed genes. After estrogen treatment, we observed increased total number of sequence-tags for H3K9ac and H3K14ac at the promoters of both estrogen-induced and estrogen-repressed genes; we also observed increased total number of sequence-tags for H3K4me3 and H3K9me3 at the estrogen-induced genes; however, we did not observe any significant change of inhibitory histone markers (H3K27me3) for either estrogen-induced or estrogen-repressed genes. The lack of repressive histone markers at the estrogen-repressed genes indicates that estrogen-mediated gene repression is not caused by, or associated with, changes in histone modification, at least not for the set of anchor genes we studied.

#### Chromatin reconfiguration

**Figure 3.**
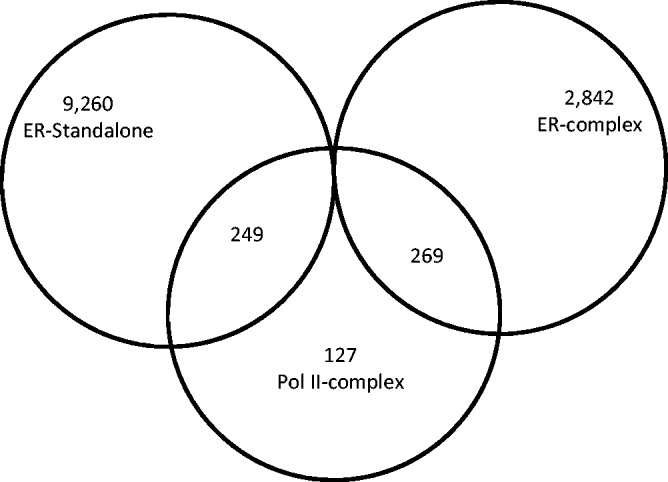
A Venn diagram illustrating the overlap between the Pol II complexes formed in the absence of ligand and the ERα complexes formed in the presence of ligand.

Because the majority of estrogen-responsive genes was enclosed in higher-order chromatin structures in the absence of estrogen, investigating the impact of estrogen treatment on these structures could shed light on their role in transcription regulation (39–45). Analyzing the results of a ChIA-PET study of ERα chromatin complexes (15) in the presence of estrogen, we identified 4293 higher-order chromatin complexes involving ERα in the presence of estrogen, as well as 10 729 ‘stand-alone’ ERα-binding events (external to a higher-order chromatin structure). Figure 3 shows the number of ERα complexes and stand-alone ERα-binding sites overlapping the 552 Pol II chromatin complexes enclosing the estrogen-responsive genes reported above. A total of 358 out of 624 estrogen-responsive genes were associated with ERα chromatin complexes after estrogen treatment. We then inferred whether the Pol II complexes in the absence of estrogen were retained or disrupted after estrogen treatment by integrating the results of Pol II ChIA-PET and ER ChIA-PET experiments performed in the absence and presence, respectively, of estrogen.

**Figure 4.**
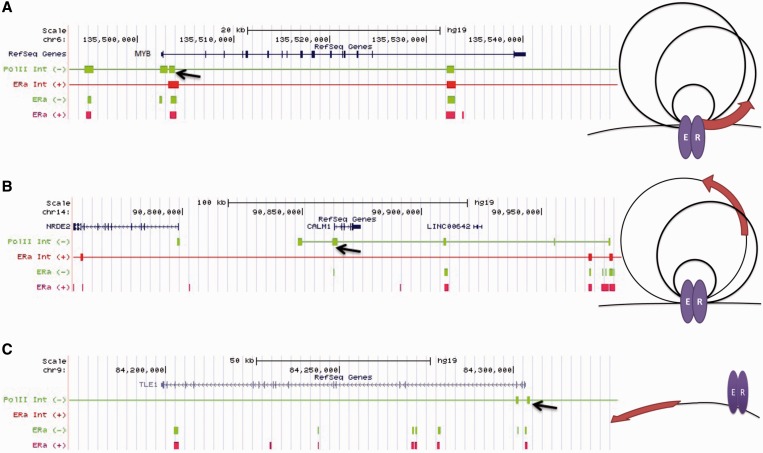
The position transition patterns of estrogen-responsive genes with respect to Pol II and ERα complexes and examples of positional transition of genes with respect to Pol II and ERα ChIA-PET complexes. (**A**) anchor-to-anchor, (**B**) anchor-to-loop, (**C**) anchor-to-stand-alone. A brown arrow represents a gene. The figure shows the positions and relationships of genes (RefSeq), Pol II complex anchor regions (Pol II Int), ERα complex anchor regions (ERα Int) and ERα ChIP-seq binding sites in the absence and presence of estrogen for three example genes: (A) anchor-to-anchor, gene: *MYB*; (B) anchor-to-loop, gene: *CALM1*; (C) anchor-to-stand-alone, gene: *TLE1*. The estrogen treatment conditions are color-coded with green (absence) and red (presence). The black arrows indicate one of Pol II anchor regions in the absence of ligand; a line in the chromatin interaction graph indicate the DNA region is involved in a chromatin complex.

We classified each estrogen-responsive gene anchored in Pol II complexes before estrogen according to its status in the ER complex after estrogen as follows: (i) Anchor-to-anchor genes: an anchor gene with respect to a Pol II complex was also an anchor gene with respect to an ERα complex. For example, Figure 4A shows that the Pol II and ERα chromatin complexes anchor at the same regions at the promoter of the *MYB* gene, and that the ERα-binding sites overlapped with the anchor regions of both Pol II and ERα complexes in the absence and presence of estrogen, indicating that the anti-Pol-II and anti-ERα antibodies had pulled down a common complex that existed before and after estrogen treatment. (ii) Anchor-to-loop: an anchor gene with respect to a Pol II complex converted to a loop gene with respect to an ERα complex. Figure 4B shows that, before estrogen treatment, an ERα ChIP-seq-binding site overlapped with one of the anchor regions of the Pol II complex (arrow) at the promoter of *CALM1*, indicating that both ERα and Pol II were involved in the chromatin complex. After estrogen treatment, a new ERα complex, which involved a broader DNA region subsuming the original Pol II complex, formed concurrent with the disappearance of the original ERα-binding site in the Pol II complex. Therefore, it can be inferred that the original Pol II chromatin complex was disrupted. (iii) Anchor-to-stand-alone: an anchor gene with respect to a Pol II complex became a stand-alone ERα gene; i.e. the gene promoter was within ±20 kb of non-interacting ERα-binding sites (15). An example of an ‘anchor-to-stand-alone’ gene, *TLE1*, is shown in Figure 4C. After estrogen treatment, the ERα-binding sites overlapping with the Pol II complex anchor are not associated with any ERα complex, suggesting that the original Pol II complex had been disrupted.

We first compared the distributions of estrogen-induced and estrogen-repressed genes, which were significantly different (Chi-square test, *P* < 10^−^^11^), among these categories. Based on the distribution of genes among the categories, we inferred that 86% (124/144) of the Pol II complexes, which formed in the absence of estrogen and contained estrogen-repressed genes, were disrupted after estrogen treatment. In comparison, the Pol II complexes containing 49% (104/213) of estrogen-induced genes were inferred to have been disrupted.

**Figure 5.**
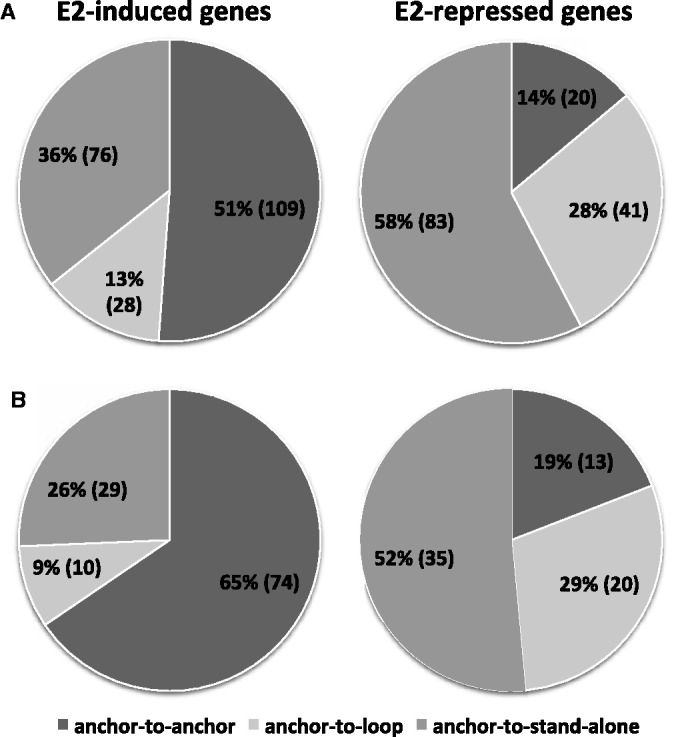
The distribution of the positional transitions of the estrogen-induced and estrogen-repressed genes. This figure shows the impact of estrogen treatment on chromatin reconfiguration (a gene within a Pol II anchor region was categorized as a loop or standalone gene with respect to ER complex after estrogen treatment) (**A**) Number of gene in each positional transitional pattern group (anchor-to-anchor, anchor-to-loop, anchor-to-stand-alone) for estrogen-induced and estrogen-repressed genes. (**B**) Positional transition pattern distribution of estrogen-responsive genes that had ERα binding in the anchor region of the original Pol II complexes in the absence of ligand.

We further studied the subset of estrogen-responsive genes with ERα inside the anchor region of the Pol II complexes in the absence of estrogen. Because these ERα were integrated in the Pol II complexes, we felt it would be interesting to investigate the impact of the binding of estrogen to ERα on the status of the Pol II complexes. After estrogen treatment, 81% (55 out of 68) of the estrogen-repressed genes in this subset underwent chromatin reconfiguration; in contrast, only 35% (39 out of 113) of estrogen-induced genes underwent chromatin reconfiguration (Figure 5B).

#### Impact of chromatin reconfiguration on transcription

To investigate the effect of chromatin reconfiguration on transcription activities, we compared transcription activities of different gene groups, e.g. ‘anchor-to-anchor’ group vs. ‘anchor-to-loop’ group, based on the GRO-seq data (16). For the estrogen-induced genes, the transcription activities were not significantly different among the anchor-to-anchor, anchor-to-loop and anchor-to-stand-alone groups in the presence of estrogen, and all subgroups showed increased transcription activity after estrogen treatment, compared with activity before estrogen treatment.

For the estrogen-repressed genes, we compared the transcription activities of the ‘anchor-to-anchor’ genes with those of the genes having undergone a chromatin configuration (pooled ‘anchor-to-loop’ and ‘anchor-to-stand-alone’ genes) in the presence of estrogen. The results revealed significantly higher (*t*-test, *P* = 0.04) transcription activities of the ‘anchor-to-anchor’ genes. Interestingly, we noted that the transcription activities of the ‘anchor-to-anchor’ genes were insensitive to estrogen treatment, whereas the genes that had undergone chromatin configuration exhibited significant changes in response to estrogen treatment (paired *t*-test for ‘anchor-to-loop’: *P* = 0.0006; ‘anchor-to-stand-alone’: *P* = 0.0024). In a further comparison between the ‘anchor-to-anchor’ genes from the estrogen-induced and estrogen-repressed groups, we found that the transcription activities were not significantly different (*t*-test, *P* = 0.45). These results indicate that, for the estrogen-repressed genes, chromatin reconfiguration had a significant impact on their transcription activities.

The discrepancies between the *de novo* transcription activities measured using GRO-seq and the steady-state mRNA levels measured using microarrays for the ‘anchor-to-anchor’ estrogen-repressed genes led to the hypothesis that these genes were affected by active posttranscriptional regulations (for example, through miRNAs). Several genome-wide profiling studies have characterized estrogen-dependent miRNAs, whose transcription is induced by estrogen (46,47). These miRNAs, including miR-22, let-7, miR-221/222, miR-18a/19b/20b and miR17-5p, were shown to negatively modulate the ERα-regulated genes after estrogen stimulation. Using the software package ComiR (28), we found that 13 of the 20 ‘anchor-to-anchor’ estrogen-repressed genes were likely targets of at least one of these miRNAs (Fisher’s *P* < 0.05; Supplementary Table S4).

#### Other TF/co-regulators

We further examined the distribution of a set of well-known ERα-partner TFs and co-regulators using the data of ChIP-seq experiments performed in MCF-7 breast cancer cells treated with estrogen (17,19,20,25,33,35,48). We examined their distribution within different subgroups of estrogen-responsive genes; results are shown in Supplementary Table S3. Interestingly, nuclear receptor coactivator 1 (SRC-1), SRC-2, SRC-3 and FoxA1 binding events were found to be enriched in the ‘anchor-to-anchor’ group of the estrogen-induced genes. This observation indicates that co-activators SRC-1, SRC-2 and SRC-3 were preferentially recruited to estrogen-induced genes.

## DISCUSSION

**Figure 6.**
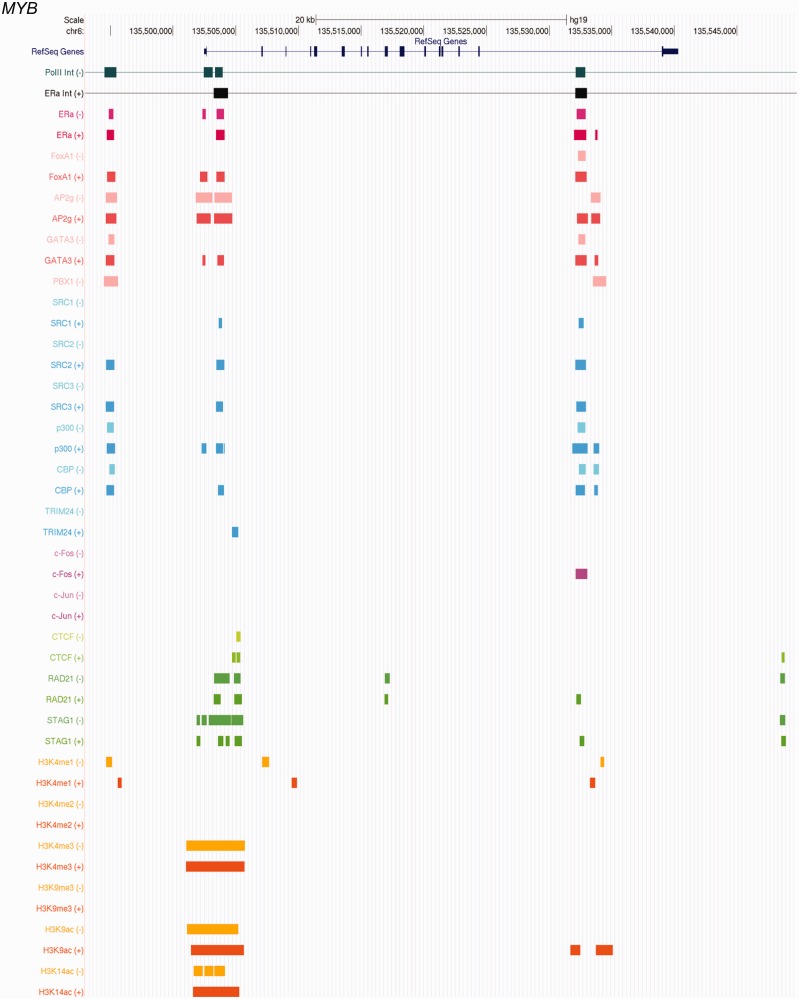
An example of integrative view of the transcription machinery at a gene. The figure shows the Pol II and ERα ChIA-PET data and ChIP-seq data in the vicinity of *MYB* gene in the absence (−) and presence (+) of estrogen. For the chromatin-interaction trace, a line indicates the DNA region is part of a chromatin structure, and a bar shows one of the anchor regions, where DNA interacts with a protein complex. For ChIP-seq data, a block indicates a DNA–protein binding site.

In this article, we present the results of an integrative analysis of multiple genome-scale data to derive a holistic view of the transcription machineries at estrogen-responsive genes in the presence and absence of estrogen. Figure 6 shows an example of such a view rendered in a genome browser format. As a resource to enable the research community to investigate different aspects of these genes in the context of chromatin structure and protein–DNA interactions, we provide a supplementary Web Site supplying the results of all estrogen-sensitive anchor genes studied in this report. Large-scale information integration enabled us to systematically dissect the data from multiple angles to reveal different mechanisms of estrogen-mediated transcription regulation, work that would not have been possible using only one or a few of the available data types. Our analyses have led to novel insights regarding the mechanisms of estrogen- and ERα-mediated gene expression regulation, creating the potential for broadly refining our understanding of the mechanisms of a large family of nuclear receptors.

In the absence of estrogen, most of the estrogen-responsive genes, both estrogen-induced and estrogen-repressed, assumed a higher-order chromatin structure that involved Pol II, ERα and ERα-pioneer factors; these promoters were also associated with active histone markers. The results indicate that the transcription machineries at these genes were well poised to respond to estrogen quickly. Interestingly, in the absence of estrogen, the genes to be induced showed active transcription initiation at TSSs but failed to elongate into gene bodies. Such transcription pauses may be due to (i) the presence of ERα co-repressors, e.g. nuclear receptor corepressor (N-CoR) or silencing mediator of retinoid acid and thyroid hormone receptor (SRMT) (49), recruited by estrogen-free ERα; or (ii) the estrogen-free ERα leading to an overall complex conformation that failed to recruit co-activators, such as SRC-1 (49), which might be required for efficient transcription elongation. These results indicate that binding of estrogen to ERα is not required for assembling transcription machineries at estrogen-responsive genes, but rather that the role of estrogen is to regulate the activity of already assembled machineries.

Through careful data integration and reasoning, we report the first evidence that the majority of estrogen-repressed genes underwent a chromatin reconfiguration after estrogen treatment that had a significant impact on the transcription activities of the genes. This leads to a hypothesis that the original Pol II complexes provided an ideal transcription environment for these genes, and that the disruption of the structures in response to estrogen impaired their transcription, thus rendering them estrogen-repressed. We hypothesize the following potential mechanisms for estrogen/ERα-mediated chromatin reconfiguration: (i) the binding of estrogen to ERα within the preexisting chromatin complexes induced conformation changes in ERα, which in turn caused the conformation changes of the overall complexes, thus disrupting the complexes; (ii) estrogen-bound ERα led to new high-affinity binding events and/or formation of new ERα-mediated complexes within a close vicinity of the preexisting Pol II complexes, and these events exerted physical distortion on the chromatin surrounding the original complexes, leading to their disruption; or (iii) a combination of the above mechanisms.

In comparison, the majority of estrogen-induced genes assumed an ‘anchor-to-anchor’ pattern after estrogen treatment, and therefore retained the active chromatin states at the TSSs. Estrogen treatment likely facilitated transcription elongation by recruiting co-activators or releasing co-repressors. Observed enrichment of co-activators SRC-1, SRC-2 and SRC-3 at the promoters of these genes supports this notion. It will be interesting to further investigate whether known ERα co-repressors, such as N-CoR or SMRT, are part of the chromatin structures formed in the absence of estrogen, and to study the impact of estrogen on chromatin localization of co-repressors. For the estrogen-induced genes that underwent chromatin reconfiguration, the originally transcription-inefficient complexes may be replaced by new ERα complexes that are transcription-favoring.

## SUPPLEMENTARY DATA

Supplementary Data are available at NAR Online.

Supplementary Data

## References

[1] Osborne CK, Schiff R, Fuqua SA, Shou J (2001). Estrogen receptor: current understanding of its activation and modulation. Clin. Cancer Res..

[2] Deroo BJ, Korach KS (2006). Estrogen receptors and human disease. J. Clin. Invest..

[3] Carroll JS, Meyer CA, Song J, Li W, Geistlinger TR, Eeckhoute J, Brodsky AS, Keeton EK, Fertuck KC, Hall GF (2006). Genome-wide analysis of estrogen receptor binding sites. Nat. Genet..

[4] Welboren WJ, Sweep FC, Span PN, Stunnenberg HG (2009). Genomic actions of estrogen receptor alpha: what are the targets and how are they regulated?. Endocr. Relat. Cancer.

[5] Leitman DC, Paruthiyil S, Yuan C, Herber CB, Olshansky M, Tagliaferri M, Cohen I, Speed TP (2012). Tissue-specific regulation of genes by estrogen receptors. Semin. Reprod. Med..

[6] Perissi V, Menini N, Cottone E, Capello D, Sacco M, Montaldo F, De Bortoli M (2000). AP-2 transcription factors in the regulation of ERBB2 gene transcription by oestrogen. Oncogene.

[7] Newman SP, Bates NP, Vernimmen D, Parker MG, Hurst HC (2000). Cofactor competition between the ligand-bound oestrogen receptor and an intron 1 enhancer leads to oestrogen repression of ERBB2 expression in breast cancer. Oncogene.

[8] Malik S, Jiang S, Garee JP, Verdin E, Lee AV, O'Malley BW, Zhang M, Belaguli NS, Oesterreich S (2010). Histone deacetylase 7 and FoxA1 in estrogen-mediated repression of RPRM. Mol. Cell. Biol..

[9] Stossi F, Likhite VS, Katzenellenbogen JA, Katzenellenbogen BS (2006). Estrogen-occupied estrogen receptor represses cyclin G2 gene expression and recruits a repressor complex at the cyclin G2 promoter. J. Biol. Chem..

[10] Oesterreich S, Deng W, Jiang S, Cui X, Ivanova M, Schiff R, Kang K, Hadsell DL, Behrens J, Lee AV (2003). Estrogen-mediated down-regulation of E-cadherin in breast cancer cells. Cancer Res..

[11] Simonis M, Klous P, Splinter E, Moshkin Y, Willemsen R, de Wit E, van Steensel B, de Laat W (2006). Nuclear organization of active and inactive chromatin domains uncovered by chromosome conformation capture-on-chip (4C). Nat. Genet..

[12] Zhao Z, Tavoosidana G, Sjolinder M, Gondor A, Mariano P, Wang S, Kanduri C, Lezcano M, Sandhu KS, Singh U (2006). Circular chromosome conformation capture (4C) uncovers extensive networks of epigenetically regulated intra- and interchromosomal interactions. Nat. Genet..

[13] Dostie J, Richmond TA, Arnaout RA, Selzer RR, Lee WL, Honan TA, Rubio ED, Krumm A, Lamb J, Nusbaum C (2006). Chromosome Conformation Capture Carbon Copy (5C): a massively parallel solution for mapping interactions between genomic elements. Genome Res..

[14] Lieberman-Aiden E, van Berkum NL, Williams L, Imakaev M, Ragoczy T, Telling A, Amit I, Lajoie BR, Sabo PJ, Dorschner MO (2009). Comprehensive mapping of long-range interactions reveals folding principles of the human genome. Science.

[15] Fullwood MJ, Liu MH, Pan YF, Liu J, Xu H, Mohamed YB, Orlov YL, Velkov S, Ho A, Mei PH (2009). An oestrogen-receptor-alpha-bound human chromatin interactome. Nature.

[16] Hah N, Danko CG, Core L, Waterfall JJ, Siepel A, Lis JT, Kraus WL (2011). A rapid, extensive, and transient transcriptional response to estrogen signaling in breast cancer cells. Cell.

[17] Joseph R, Orlov YL, Huss M, Sun W, Kong SL, Ukil L, Pan YF, Li G, Lim M, Thomsen JS (2010). Integrative model of genomic factors for determining binding site selection by estrogen receptor-alpha. Mol. Syst. Biol..

[18] Welboren WJ, van Driel MA, Janssen-Megens EM, van Heeringen SJ, Sweep FC, Span PN, Stunnenberg HG (2009). ChIP-Seq of ERalpha and RNA polymerase II defines genes differentially responding to ligands. EMBO J..

[19] Schmidt D, Schwalie PC, Ross-Innes CS, Hurtado A, Brown GD, Carroll JS, Flicek P, Odom DT (2010). A CTCF-independent role for cohesin in tissue-specific transcription. Genome Res..

[20] Hurtado A, Holmes KA, Ross-Innes CS, Schmidt D, Carroll JS (2011). FOXA1 is a key determinant of estrogen receptor function and endocrine response. Nat. Genet..

[21] Tang Q, Chen Y, Meyer C, Geistlinger T, Lupien M, Wang Q, Liu T, Zhang Y, Brown M, Liu XS (2011). A comprehensive view of nuclear receptor cancer cistromes. Cancer Res..

[22] Jagannathan V, Robinson-Rechavi M (2011). Meta-analysis of estrogen response in MCF-7 distinguishes early target genes involved in signaling and cell proliferation from later target genes involved in cell cycle and DNA repair. BMC Syst. Biol..

[23] Li G, Ruan X, Auerbach RK, Sandhu KS, Zheng M, Wang P, Poh HM, Goh Y, Lim J, Zhang J (2012). Extensive promoter-centered chromatin interactions provide a topological basis for transcription regulation. Cell.

[24] Kuttippurathu L, Hsing M, Liu Y, Schmidt B, Maskell DL, Lee K, He A, Pu WT, Kong SW (2011). CompleteMOTIFs: DNA motif discovery platform for transcription factor binding experiments. Bioinformatics.

[25] Tan SK, Lin ZH, Chang CW, Varang V, Chng KR, Pan YF, Yong EL, Sung WK, Cheung E (2011). AP-2gamma regulates oestrogen receptor-mediated long-range chromatin interaction and gene transcription. EMBO J..

[26] Zhang Y, Liu T, Meyer CA, Eeckhoute J, Johnson DS, Bernstein BE, Nusbaum C, Myers RM, Brown M, Li W (2008). Model-based analysis of ChIP-Seq (MACS). Genome Biol..

[27] Bonn S, Zinzen RP, Girardot C, Gustafson EH, Perez-Gonzalez A, Delhomme N, Ghavi-Helm Y, Wilczynski B, Riddell A, Furlong EE (2012). Tissue-specific analysis of chromatin state identifies temporal signatures of enhancer activity during embryonic development. Nat. Genet..

[28] Coronnello C, Hartmaier R, Arora A, Huleihel L, Pandit KV, Bais AS, Butterworth M, Kaminski N, Stormo GD, Oesterreich S (2012). Novel modeling of combinatorial miRNA targeting identifies SNP with potential role in bone density. PLoS Comput. Biol..

[29] Favorov A, Mularoni L, Cope LM, Medvedeva Y, Mironov AA, Makeev VJ, Wheelan SJ (2012). Exploring massive, genome scale datasets with the GenometriCorr package. PLoS Comput. Biol..

[30] Pruitt KD, Tatusova T, Maglott DR (2007). NCBI reference sequences (RefSeq): a curated non-redundant sequence database of genomes, transcripts and proteins. Nucleic Acids Res..

[31] Dillon N (2006). Gene regulation and large-scale chromatin organization in the nucleus. Chromosome Res..

[32] Magnani L, Ballantyne EB, Zhang X, Lupien M (2011). PBX1 genomic pioneer function drives ERalpha signaling underlying progression in breast cancer. PLoS Genet..

[33] Kong SL, Li G, Loh SL, Sung WK, Liu ET (2011). Cellular reprogramming by the conjoint action of ERalpha, FOXA1, and GATA3 to a ligand-inducible growth state. Mol. Syst. Biol..

[34] Zhou VW, Goren A, Bernstein BE (2011). Charting histone modifications and the functional organization of mammalian genomes. Nat. Rev. Genet..

[35] Tsai WW, Wang Z, Yiu TT, Akdemir KC, Xia W, Winter S, Tsai CY, Shi X, Schwarzer D, Plunkett W (2010). TRIM24 links a non-canonical histone signature to breast cancer. Nature.

[36] Core LJ, Waterfall JJ, Lis JT (2008). Nascent RNA sequencing reveals widespread pausing and divergent initiation at human promoters. Science.

[37] Seila AC, Calabrese JM, Levine SS, Yeo GW, Rahl PB, Flynn RA, Young RA, Sharp PA (2008). Divergent transcription from active promoters. Science.

[38] Seila AC, Core LJ, Lis JT, Sharp PA (2009). Divergent transcription: a new feature of active promoters. Cell Cycle.

[39] Spilianakis CG, Flavell RA (2004). Long-range intrachromosomal interactions in the T helper type 2 cytokine locus. Nat. Immunol..

[40] Tsytsykova AV, Rajsbaum R, Falvo JV, Ligeiro F, Neely SR, Goldfeld AE (2007). Activation-dependent intrachromosomal interactions formed by the TNF gene promoter and two distal enhancers. Proc. Natl Acad. Sci. USA.

[41] Drissen R, Palstra RJ, Gillemans N, Splinter E, Grosveld F, Philipsen S, de Laat W (2004). The active spatial organization of the beta-globin locus requires the transcription factor EKLF. Genes Dev..

[42] Vakoc CR, Letting DL, Gheldof N, Sawado T, Bender MA, Groudine M, Weiss MJ, Dekker J, Blobel GA (2005). Proximity among distant regulatory elements at the beta-globin locus requires GATA-1 and FOG-1. Mol. Cell.

[43] Jing H, Vakoc CR, Ying L, Mandat S, Wang H, Zheng X, Blobel GA (2008). Exchange of GATA factors mediates transitions in looped chromatin organization at a developmentally regulated gene locus. Mol. Cell.

[44] Levasseur DN, Wang J, Dorschner MO, Stamatoyannopoulos JA, Orkin SH (2008). Oct4 dependence of chromatin structure within the extended Nanog locus in ES cells. Genes Dev..

[45] Carroll JS, Liu XS, Brodsky AS, Li W, Meyer CA, Szary AJ, Eeckhoute J, Shao W, Hestermann EV, Geistlinger TR (2005). Chromosome-wide mapping of estrogen receptor binding reveals long-range regulation requiring the forkhead protein FoxA1. Cell.

[46] Castellano L, Giamas G, Jacob J, Coombes RC, Lucchesi W, Thiruchelvam P, Barton G, Jiao LR, Wait R, Waxman J (2009). The estrogen receptor-alpha-induced microRNA signature regulates itself and its transcriptional response. Proc. Natl Acad. Sci. USA.

[47] Bhat-Nakshatri P, Wang G, Collins NR, Thomson MJ, Geistlinger TR, Carroll JS, Brown M, Hammond S, Srour EF, Liu Y (2009). Estradiol-regulated microRNAs control estradiol response in breast cancer cells. Nucleic Acids Res..

[48] Zwart W, Theodorou V, Kok M, Canisius S, Linn S, Carroll JS (2011). Oestrogen receptor-co-factor-chromatin specificity in the transcriptional regulation of breast cancer. EMBO J..

[49] Merrell KW, Crofts JD, Smith RL, Sin JH, Kmetzsch KE, Merrell A, Miguel RO, Candelaria NR, Lin CY (2011). Differential recruitment of nuclear receptor coregulators in ligand-dependent transcriptional repression by estrogen receptor-alpha. Oncogene.

