# Supplementary material for: Estrogen represses gene expression through reconfiguring chromatin structures
Source: Nucleic Acids Res. 2013 Jul 1;41(17):8061–71. doi: 10.1093/nar/gkt586 (PMC3783169; doi:10.1093/nar/gkt586)
Supplement: Supplementary Data [file supp_41_17_8061__index.html]

Estrogen represses gene expression through reconfiguring chromatin structures — Estrogen represses gene expression through reconfiguring chromatin structures — Supplementary Data 

# Estrogen represses gene expression through reconfiguring chromatin structures

## 

files

**Files in this Data Supplement:**

- Supplementary Data - docx file
