# Supplementary material for: Estrogen represses gene expression through reconfiguring chromatin structures
Source: Nucleic Acids Res. 2013 Jul 1;41(17):8061–71. doi: 10.1093/nar/gkt586 (PMC3783169; doi:10.1093/nar/gkt586)
Supplement: Supplementary Data [file supp_gkt586_nar-01035-m-2013-File008.docx]

# Estrogen Represses Gene Expression through Reconfiguring Chromatin Structures

Hatice Ulku Osmanbeyoglu^1^, Kevin N. Lu^2^, Steffi Oesterreich^3, 4^, Roger S. Day^1^, Panayiotis V. Benos^5^, Claudia Coronnello^5, 6^, and Xinghua Lu^1§^

# Supplementary website

A supplementary website that renders the figures for all estrogen-sensitive genes, containing all chromatin structures and binding events of TFs in the format of UCSC genome browser figure, can be accessed at the following URL: <http://chromatin-structure-of-estrogen-sensitive-genes.dbmi.pitt.edu/>

# Supplementary Figures

## Supplementary Figure 1 – Number of overlapping pioneer factor binding sites inside Pol II complexes in the absence of ligand

The Venn diagram shows the number of overlapping pioneer factor binding sites (at least one bp overlap) inside the Pol II complex regions (552) where identified estrogen-responsive anchor genes reside


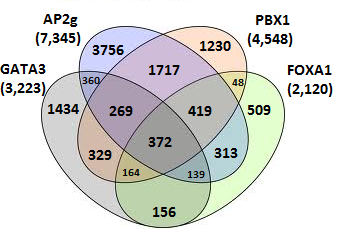


## Supplementary Figure 2 – Number of overlapping pioneer factor binding sites with ERα inside Pol II complexes in the absence of ligand

The Venn diagram shows the number of overlapping pioneer factor binding sites with ERα binding sites (at least one bp overlap) inside the Pol II complex regions (552) containing estrogen-responsive genes

#
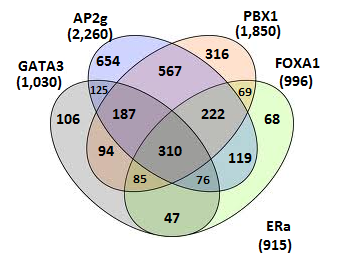
Supplementary Tables

## Supplementary Table 1 – Genes induced or repressed by estrogens

| **Estrogen Induced Genes** | | **Estrogen Repressed genes** | |
| --- | --- | --- | --- |
| ET |  | | F11B |
| CA12 |  | | ID3 |
| IGFBP4 |  | | EFNA1 |
| MYBL1 |  | | IL1R1 |
| HSPB8 |  | | ENC1 |
| WWC1 |  | | RNF43 |
| SERPINB9 |  | | SOCS2 |
| EGR3 |  | | NUAK1 |
| STC2 |  | | ID1 |
| RAPGEFL1 |  | | DDIT4 |
| KRT13 |  | | BTG2 |
| NRIP1 |  | | RWDD2A |
| PGR |  | | SH3BP4 |
| SLC22A5 |  | | NACC2 |
| MYB |  | | GTF2IRD1 |
| SLC7A5 |  | | PIK3R3 |
| RAB31 |  | | FAM115A |
| MYC |  | | ARNT2 |
| SIAH2 |  | | SIDT1 |
| RASGRP1 |  | | EGLN3 |
| ASB13 |  | | CREB3L1 |
| FOS |  | | KITLG |
| MAP6D1 |  | | TLE1 |
| TFF1 |  | | NCOA3 |
| OLFML3 |  | | BMP4 |
| LRIG1 |  | | VTCN1 |
| TIAM1 |  | | PLEKHF2 |
| SLC19A2 |  | | BTG1 |
| FZD7 |  | | MKNK2 |
| PTGES |  | | ZDHHC7 |
| JARID2 |  | | KIAA0513 |
| KDM4B |  | | ITGB6 |
| KLF4 |  | | PXN |
| PLOD2 |  | | RBMS1 |
| SLC9A3R1 |  | | PPM1H |
| PEX11A |  | | RAB20 |
| FHL2 |  | | SYTL2 |
| FAM102A |  | | TRAFD1 |
| SLC2A1 |  | | TRAM2 |
| CCDC88C |  | | PDLIM5 |
| PPIF |  | | RXRA |
| TFF2 |  | | TCF7L2 |
| CBFA2T3 |  | | ACAA2 |
| PODXL |  | | B4GALT1 |
| FADS1 |  | | GALNT10 |
| RHOBTB3 |  | | DEGS1 |
| OPN3 |  | | C19orf21 |
| MREG |  | | HOXB7 |
| SVIL |  | | KLF6 |
| ADCY1 |  | | MARCKS |
| NOC3L |  | | KIAA1609 |
| WISP2 |  | | PLEKHF1 |
| RGS10 |  | | EDN1 |
| UGCG |  | | SUOX |
| CCND1 |  | | CLDN9 |
| MED13L |  | | PMEPA1 |
| ADCY9 |  | | NRBP1 |
| PRSS23 |  | | TTC9 |
| ELF1 |  | | PAWR |
| ABHD2 |  | | SLC2A10 |
| RHOBTB1 |  | | PRSS8 |
| ANXA9 |  | | SRCAP |
| GAB2 |  | | ATXN1 |
| AMMECR1 |  | | CDH3 |
| SMOX |  | | CLDN4 |
| FAM63A |  | | RND3 |
| HEG1 |  | | ORAI3 |
| SLC25A24 |  | | TRPS1 |
| TGFA |  | | TRIB1 |
| HEY2 |  | | PPL |
| SEMA3G |  | | SPRED2 |
| DHRS2 |  | | VGLL4 |
| CHD9 |  | | DOK4 |
| FOXC1 |  | | MEX3D |
| B3GALNT1 |  | | GPR126 |
| CYR61 |  | | RARG |
| FKBP4 |  | | CDKN2D |
| CELSR2 |  | | SPDEF |
| DTL |  | | CD55 |
| CARD10 |  | | TBX3 |
| MICAL2 |  | | IGFBP5 |
| NXT1 |  | | RMND1 |
| DKC1 |  | | TMEM135 |
| SBNO2 |  | | MAZ |
| RCL1 |  | | DUSP4 |
|  |  | |  |
| CHSY1 |  | | RMND5B |
| SLC6A6 |  | | ELF3 |
| HK2 |  | | ATP9A |
| HLA-DRB1 |  | | FKBP8 |
| DYNLT3 |  | | GRB14 |
| SLC22A4 |  | | FFAR2 |
| OSGIN1 |  | | SGMS1 |
| TBKBP1 |  | | CERK |
| SLC25A32 |  | | KCNG1 |
| MAP4K3 |  | | TSC22D3 |
| DNAJB9 |  | | ATP2B1 |
| FAM134B |  | | SLC2A6 |
| BHLHE41 |  | | BCAR3 |
| PNO1 |  | | TEAD3 |
| SPINK4 |  | | EPHB3 |
| UNC119 |  | | SEMA4C |
| TIPIN |  | | LCAT |
| ADHFE1 |  | | ATP2C2 |
| SIDT2 |  | | HEXIM1 |
| CA2 |  | | GAS2L1 |
| GLA |  | | OSBPL10 |
| PPFIA4 |  | | ZNF821 |
| KCNF1 |  | | STK38L |
| FUT4 |  | | ZHX2 |
| ADORA1 |  | | RBL2 |
| SLC16A6 |  | | KRT7 |
| OSTF1 |  | | SREBF1 |
| RHOD |  | | BAMBI |
| OGFOD1 |  | | FAM110B |
| PTRH2 |  | | TRAF4 |
| NMD3 |  | | TIMP3 |
| CYP1B1 |  | | TNFRSF21 |
| CALCR |  | | PACSIN2 |
| ZNF267 |  | | SP2 |
| DNAJC2 |  | | TFAP2A |
| NCOR2 |  | | SERPINB1 |
| CDC42EP1 |  | | SSH3 |
| EXOSC2 |  | | PACSIN3 |
| BLVRB |  | | KIAA0247 |
| SLC27A2 |  | | SPATA20 |
| SEC14L2 |  | | THBS1 |
| RPL37A |  | | OSR2 |
| PITX1 |  | | FA2H |
| GADD45B |  | | PIK3C2B |
| UMPS |  | | IFI27 |
|  |  | |  |
| SNAPC1 |  | | PPP1R13L |
| GEM |  | | NAMPT |
| WFS1 |  | | MB |
| MYOF |  | | NUAK2 |
| NIP7 |  | | RAB35 |
| SLC30A1 |  | | SIK1 |
| CHPT1 |  | | ALDH3B2 |
| STK17A |  | | ITPKB |
| TMEM12B |  | | SYNGR3 |
| PAPSS2 |  | | KLF5 |
| PRKAG2 |  | | SOX13 |
| IL6ST |  | | PTCH1 |
| SLC25A36 |  | | ATP2A3 |
| CISH |  | |  |
| RRP12 |  | |  |
| VEGFA |  | |  |
| MINA |  | |  |
| PPRC1 |  | |  |
| FLNB |  | |  |
| ARL3 |  | |  |
| ENDOD1 |  | |  |
| NUFIP1 |  | |  |
| LARP4 |  | |  |
| INPP5A |  | |  |
| DNMBP |  | |  |
| TFAM |  | |  |
| TFRC |  | |  |
| CTSD |  | |  |
| SOX3 |  | |  |
| BACE1 |  | |  |
| DHRS3 |  | |  |
| SH3BP5 |  | |  |
| EN2 |  | |  |
| TIMM9 |  | |  |
| GINS2 |  | |  |
| BAG1 |  | |  |
| THOC5 |  | |  |
| PPAN |  | |  |
| UTP3 |  | |  |
| SHB |  | |  |
| PMM2 |  | |  |
| EFHD2 |  | |  |
| DHCR7 |  | |  |
| SP3 |  | |  |
| ABTB2 |  | |  |
|  |  | |  |
| BCL11B |  | |  |
| ZNF259 |  | |  |
| SUB1 |  | |  |
| CCNE1 |  | |  |
| ATP2A2 |  | |  |
| SLC7A6 |  | |  |
| CRKL |  | |  |
| LRRFIP2 |  | |  |
| FOXK2 |  | |  |
| TNS1 |  | |  |
| EIF2S2 |  | |  |
| MAT2A |  | |  |
| C5orf28 |  | |  |
| NOL11 |  | |  |
| C10orf137 |  | |  |
| GNL2 |  | |  |
| ECE1 |  | |  |
| RFC3 |  | |  |
| HSPA4 |  | |  |
| MSMB |  | |  |
| CSE1L |  | |  |
| RAB11FIP1 |  | |  |
| ABCD3 |  | |  |
| ANKRD46 |  | |  |
| PIP4K2A |  | |  |
| IPPK |  | |  |
| ZNF146 |  | |  |
| CLASP2 |  | |  |
| SERBP1 |  | |  |
| ETF1 |  | |  |
| NAT10 |  | |  |
| TBC1D30 |  | |  |
| DNAJA1 |  | |  |
| CAND1 |  | |  |
| CCNL1 |  | |  |
| NBN |  | |  |
| DDX47 |  | |  |
| SEC62 |  | |  |

## Supplementary Table 2 – Summary of datasets used in the study

| Name | Usage | Factors (E2 treatment time) | Datasets | Reference |
| --- | --- | --- | --- | --- |
| Microarray | Identification of early estrogen-responsive genes | (3hr/4hr) | GSE3834, GSE9936, GSE11324, GSE5840 | ([3](#_ENREF_3),[22](#_ENREF_22),[50-52](#_ENREF_50)) |
| GRO-seq | Instantaneous transcription activity | (40min) | GSE27463 | ([16](#_ENREF_16)) |
| ChIP-seq | Whole-genome mapping of protein-DNA interactions | ERα (45min/1hr/3hr), Pol II(1hr), FoxA1(45min/3hr), AP2γ(45min), PBX1, GATA3(45min), CTCF(45min), RAD21(45min), STAG1(45min), SRC-1(3hr), SRC-2(3hr), SRC-3(3hr), TRIM24(6hr),  c-Fos(3hr), c-Jun(3hr), p300(3h), CBP(3hr) | GSE14664, GSE26831, GSE24166, GSE23893, GSE28007, GSE23852, GSE25710, GSE25021 | ([17-20](#_ENREF_17),[27-30](#_ENREF_27),[32](#_ENREF_32),[46](#_ENREF_46)) |
|  | Genome-wide histone modification patterns | H3K4me1 (3hr),  H3K4me2 (6hr),  H3K4me3 (3hr),  H3K9me3 (3hr),  H3K9ac (3hr),  H3K14ac (3hr) | GSE23701, GSE24166 | ([17](#_ENREF_17),[32](#_ENREF_32)) |
| ChIA-PET | 3D chromosomal structure around a protein | ERα(45min), Pol II | GSE33664, GSE39495 | ([15](#_ENREF_15),[24](#_ENREF_24)) |

## Supplementary Table 3 – Summary of association between histone marks and estrogen-responsive gene promoters. A positive correlation coefficient indicates that the marker is enriched in the region of interest, whereas a negative one indicates that the marker is depleted in the region of interest.

| Spatial Correlation Coefficient | | | | |
| --- | --- | --- | --- | --- |
|  | E2 - | | E2 + | |
| Histone marks | E2-induced | E2-repressed | E2-induced | E2-repressed |
| H3K9ac | 0.60** | 0.50** | 0.65** | 0.51** |
| H3K14ac | 0.46** | 0.40** | 0.53** | 0.48** |
| H3K4me1 | 0.15** | 0.10** | 0.23** | 0.21** |
| H3K4me2 | -0.06* | 0.02 | -0.02 | -0.01 |
| H3K4me3 | 0.75** | 0.64** | 0.68** | 0.65** |
| H3K9me3 | -0.01 | -0.01 | -0.04 | 0.00 |
| H3K27me3 | -0.03 | 0.03 | 0.00 | 0.00 |

P-value < 0.002 **, 0.05<P-value $\leq$0.002 *

| **Supplementary Table 4 - Summary of miRNA:target binding probabilities** The table shows the ComiR scores obtained for each of the 20 anchor-to-anchor genes (1st and 2nd columns) and Estrogen receptor alpha gene (ERα) versus 8 ERa induced miRNAs (3rd to 10th columns). A gene is predicted as target at a miRNA if the relative ComiR score is greather than 0.8 (red cells). The 13 genes predicted as target are the highlighted with blue ones. ERα is predicted as target of 5 of the considered miRNAs. | | | | | | | | | |
| --- | --- | --- | --- | --- | --- | --- | --- | --- | --- |
|  |  | **ComiR scores** | | | | | | | |
| **GENE_ID** | **ENSEMBL_ID** | **hsa.let.7f.5p** | **hsa.miR.17.5p** | **hsa.miR.18a.3p** | **hsa.miR.19b.2.5p** | **hsa.miR.20b.5p** | **hsa.miR.221.5p** | **hsa.miR.222.5p** | **hsa.miR.22.3p** |
| ATXN1 | ENSG00000124788 | 0.8663 | 0.9441 | 0.8881 | 0.3078 | 0.944 | 0.8211 | 0.855 | 0.8511 |
| Thbs1 | ENSG00000137801 | 0.9219 | 0.2884 | 0.2724 | 0.825 | 0.29 | 0.2854 | 0.8529 | 0.2565 |
| TFAP2A | ENSG00000137203 | 0.916 | 0.2574 | 0.235 | 0.8664 | 0.2606 | 0.2326 | 0.2936 | 0.4168 |
| Hexim1 | ENSG00000186834 | 0.2447 | 0.2717 | 0.253 | 0.817 | 0.2719 | 0.1827 | 0.244 | 0.9067 |
| EDN1 | ENSG00000078401 | 0.9039 | 0.1723 | 0.2429 | 0.8312 | 0.1703 | 0.206 | 0.239 | 0.2108 |
| B4GALT1 | ENSG00000086062 | 0.2768 | 0.2931 | 0.7984 | 0.8267 | 0.2906 | 0.8952 | 0.2792 | 0.4701 |
| TEAD3 | ENSG00000007866 | 0.7823 | 0.2488 | 0.8832 | 0.1786 | 0.2493 | 0.782 | 0.2526 | 0.8165 |
| ZHX2 | ENSG00000178764 | 0.2635 | 0.872 | 0.221 | 0.1729 | 0.8682 | 0.2196 | 0.2118 | 0.1726 |
| PMEPA1 | ENSG00000124225 | 0.2716 | 0.3805 | 0.8716 | 0.8375 | 0.3803 | 0.8168 | 0.8459 | 0.829 |
| ppl | ENSG00000118898 | 0.1974 | 0.2117 | 0.2064 | 0.721 | 0.2086 | 0.8433 | 0.2487 | 0.2196 |
| BAMBI | ENSG00000095739 | 0.1947 | 0.8348 | 0.1566 | 0.195 | 0.836 | 0.1561 | 0.7635 | 0.1558 |
| elf3 | ENSG00000163435 | 0.1839 | 0.2626 | 0.2549 | 0.8148 | 0.2624 | 0.7923 | 0.8329 | 0.2394 |
| socs2 | ENSG00000120833 | 0.2245 | 0.2359 | 0.1707 | 0.2373 | 0.2433 | 0.168 | 0.8072 | 0.7975 |
| CLDN4 | ENSG00000189143 | 0.2214 | 0.1663 | 0.7686 | 0.1655 | 0.1661 | 0.7218 | 0.2317 | 0.2047 |
| SPDEF | ENSG00000124664 | 0.2344 | 0.2044 | 0.7633 | 0.1552 | 0.2028 | 0.7504 | 0.1566 | 0.1562 |
| bcar3 | ENSG00000137936 | 0.1859 | 0.1833 | 0.1533 | 0.1836 | 0.1886 | 0.2018 | 0.7507 | 0.2043 |
| RXRA | ENSG00000186350 | 0.1969 | 0.1521 | 0.7372 | 0.1533 | 0.1518 | 0.2081 | 0.1525 | 0.152 |
| RMND5B | ENSG00000145916 | 0.2257 | 0.1915 | 0.2096 | 0.1574 | 0.1881 | 0.1957 | 0.1564 | 0.1975 |
| Krt7 | ENSG00000135480 | 0.1909 | 0.1454 | 0.144 | 0.1423 | 0.1449 | 0.2174 | 0.1447 | 0.142 |
| LCAT | ENSG00000213398 | 0.1443 | 0.1431 | 0.1411 | 0.142 | 0.1438 | 0.142 | 0.1741 | 0.1414 |
| ERα | ENSG00000091831 | 0.3211 | 0.9451 | 0.9029 | 0.3102 | 0.9451 | 0.2825 | 0.8554 | 0.9467 |

## Supplementary Table 5 – The number of genes having TF and co-regulators binding sites within ±20 kb from their TSSs in each transition group

The table shows the distribution of binding sites among the 6 gene groups. Statistical significance was determined using chi-square tests. *P-*values were reported with Bonferroni correction. A number that is significantly deviated from the expected value (residual values greater than +2 and less than -2) is indicated with ‘+’ and ‘-’ respectively.

|  | estrogen-induced genes | | | estrogen-repressed genes | | | Adj. *P* |
| --- | --- | --- | --- | --- | --- | --- | --- |
| TF/  co-regulator | anchor-to-anchor (n=109) | anchor-to-loop (n=28) | anchor-to-SA (n=78) | anchor-to-anchor (n=20) | anchor-to-loop (n=41) | anchor-to-SA (n=83) |  |
| SRC-1 | 45 (+) | 0 (-) | 5 | 1 | 0 (-) | 0 (-) | *<10^-17^* |
| P300 | 93 | 6 (-) | 52 | 19 | 25 | 68 | *<10^-8^* |
| SRC-3 | 69 (+) | 2 (-) | 21 | 8 | 7 (-) | 24 | *<10^-7^* |
| SRC-2 | 74 (+) | 5 (-) | 29 | 14 | 9 (-) | 26 | *<10^-7^* |
| CBP | 92 | 8 (-) | 51 | 18 | 23 | 60 | *<10^-5^* |
| FoxA1 | 65 (+) | 4 (-) | 18 (-) | 9 | 12 | 27 | *<10^-4^* |
| AP2γ | 94 | 15 | 55 | 17 | 36 | 67 | 0.025 |
| TRIM24 | 75 | 16 | 36 | 11 | 31 | 61 | 0.058 |
| CTCF | 89 | 16 | 53 | 18 | 36 | 59 | 0.126 |
| Fos | 42 | 5 | 17 | 9 | 9 | 26 | 0.649 |
| RAD21 | 100 | 20 | 63 | 19 | 37 | 70 | 0.731 |
| GATA3 | 44 | 7 | 27 | 11 | 17 | 37 | 1.000 |
| STAG1 | 100 | 22 | 69 | 20 | 38 | 72 | 1.000 |
| c-Jun | 19 | 5 | 11 | 5 | 4 | 10 | 1.000 |
